# Antimicrobial Potential of Essential Oils from Cerrado Plants against Multidrug−Resistant Foodborne Microorganisms

**DOI:** 10.3390/molecules25143296

**Published:** 2020-07-21

**Authors:** Genilson Silva de Jesus, Ana Camila Micheletti, Rafael Gonçalves Padilha, Jessica de Souza de Paula, Flavio Macedo Alves, Cassia Rejane Brito Leal, Fernanda Rodrigues Garcez, Walmir Silva Garcez, Nidia Cristiane Yoshida

**Affiliations:** 1 Institute of Chemistry, Universidade Federal de Mato Grosso do Sul, Av. Senador Filinto Müller 1555, Campo Grande MS 79074460, Brazil; quimicagenilson@gmail.com (G.S.d.J.); anamicheletti@gmail.com (A.C.M.); rafael.gp13@hotmail.com (R.G.P.); depaula.biobach@gmail.com (J.d.S.d.P.); fernandargarcez@gmail.com (F.R.G.); walmir.garcez@ufms.br (W.S.G.); 2 Institute of Biosciences, Universidade Federal de Mato Grosso do Sul, Av. Costa e Silva s/n, Campo Grande MS 79070900, Brazil; flaurace@yahoo.com.br; 3 Faculty of Veterinary Medicine and Animal Science, Universidade Federal de Mato Grosso do Sul, Av. Senador Filinto Müller 2443, Campo Grande MS 79070900, Brazil; cassia.leal@ufms.br

**Keywords:** *Chromolaena squalida*, *Myrsine guianensis*, *Matayba guianensis*, *Campomanesia sessiliflora*, antibacterial, foodborne diseases

## Abstract

Foodborne pathogens are a real public health concern in an escalating antimicrobial resistance scenario. Natural products represent a promising source of bioactive molecules, and essential oils have attracted much attention due to their myriad of biological properties, including antibacterial activities. In this context, essential oils obtained from the leaves of *Chromolaena squalida*, *Campomanesia sessiliflora, Myrsine guianensis, Matayba guianensis, Siparuna guianensis, Ocotea minarum* and *Endlicheria paniculata*—species from the Cerrado biome of Midwest Brazil—were extracted and evaluated for their antibacterial activity against a panel of four standard and three clinical multidrug−resistant bacterial strains. All tested oils showed moderate to good activity against at least four bacterial strains, including *Salmonella* Typhi and oxacillin−resistant *Staphylococcus.* The essential oils from *C. squalida*, *C. sessiliflora*, *My. guianensis* and *Ma. guianensis* showed strong inhibition of clinical *Staphylococcus* strains, which cause bovine mastitis and are related to milk−borne diseases. Their chemical profiles were investigated by gas chromatography coupled to mass spectrometry (GC/MS), which revealed a predominance of mono− and sesquiterpene hydrocarbons, some of which with well−known antimicrobial properties. The essential oil from Cerrado plants proved active against resistant Gram−positive and Gram−negative bacteria, revealing their potentialities for the development of new alternative agents to prevent the spreading of resistant bacterial contamination.

## 1. Introduction

Foodborne diseases comprise a large group of illness that result from the consumption of contaminated food or water. The diseases caused by foodborne microorganisms are considered a major concern in global public health, as they can cause significant losses in society and affect the economy, due to treatment, hospitalization and epidemiological investigation costs [1,2,3]. According to the World Health Organization, the estimated number of cases of foodborne diseases is 600 million and 420 thousand deaths each year, which means that 1 in 10 people become ill from consuming contaminated food [2]. Although digestive manifestations are usually mild and self−limited, unsafe foods containing harmful bacteria, viruses, parasites or chemical substances, cause more than 200 diseases—ranging from diarrhea to cancer [2,4].

Several factors favor the increase in foodborne diseases, such as population growth, the need for large−scale food production and changes in eating habits (e.g., increasing the protein intake). The situation is of great concern, mainly due to difficulties in controlling contamination of animals and animal origin products, as they involve multiple factors, such as water, fauna, soil, slaughter and processing practices and storage, among others [5].

The most common foodborne disease worldwide is food poisoning caused by *Staphylococcus*, considered one of the main pathogens responsible for disease outbreaks related to the consumption of contaminated food [6]. It can be found in several foods, but milk and its derivatives are among the main sources of contamination, since they are good substrates for the growth of different *Staphylococcus* types and production of enterotoxins [6]. Some studies have shown that these staphylococcal enterotoxins can retain their biological activities even after the pasteurization process [7,8]. In addition, this bacterium causes bovine mastitis (inflammation of the mammary gland in the udder of dairy cows), leading to a reduction in milk quality and yield, which causes considerable economic losses in livestock [7]. These economic losses profoundly affect the economy of countries like Brazil, which is an important contributor to the dairy and meat market, as it leads the ranking of countries with the largest cattle herd (214.7 million head) [9] and is the fifth country with the highest milk production [10].

One of the main microorganisms responsible for bovine mastitis is methicillin−resistant *Staphylococcus aureus* (MRSA). The emergence of multidrug−resistant pathogenic bacteria is a public health concern, as it restricts or extinguishes the availability of the existing antimicrobials [11]. Therefore, it is essential to continuously search for new compounds with antimicrobial activity, especially against multidrug−resistant bacteria, to mitigate this problem [12].

Plant natural products represent an important source of bioactive molecules, since these organisms produce a myriad of complex and structurally diverse chemical compounds, with great pharmacological potentials [12,13]. Essential oils (EOs) are produced by plants and act in the defense against herbivores, and infections caused by microorganisms, as well as attracting pollinators, and in the plant–plant or plant–insect interactions [14,15]. In general, EOs consist of mixtures of 20 to 60 compounds, most of them belonging to the class of terpenoids (mono−, sesqui− and diterpenes) and their oxygenated and aromatic derivatives, in addition to aliphatic acid esters terpenoids and phenolic compounds (usually C_6_−C_3_ derivatives) [16].

In recent years, there has been a growing interest in the potential use of EOs in the food and cosmetics industries. Although EOs are often used in the industry as flavoring agents, these natural products possess a broad range of antimicrobial properties for food preservation. For instance, some EOs, alone or in combination with commercial antibiotics, have shown promising results against multi−resistant bacteria [17,18]. In addition, several studies have reported the activity of EOs against pathogens that cause foodborne diseases, suggesting their applications in the food industry [16].

Brazilian flora, enclosed in biomes such as Pantanal and Cerrado, represents one of the highest potentials concerning biological diversity. The Cerrado biome, one of the largest Brazilian ecosystems, covers about 21% of the Brazilian territory and houses roughly 12,000 species of plants [19], ca. 30% endemic [20]. However, its extensive degradation and lack of sustainable management, which has been intensified in recent years, have caused severe losses in Cerrado’s biodiversity, reinforcing its position as a world hotspot for the conservation of biodiversity, since it presents circa one−third of the biological diversity of Brazil and 5% of the globe [21,22]. Although several bioactive molecules have been identified from plants of this biome [23], the Cerrado flora still offers a wide biodiversity of species that remains unexplored. Therefore, the Cerrado consists of a promising source of natural products to be investigated regarding their potential for the prevention/treatment of bacterial infections.

In the face of our rich biodiversity and aiming at finding promising bioactive natural products for food preservation, the present work describes the evaluation of the antimicrobial potential of the essential oils from seven native plants of the Brazilian Cerrado against foodborne diseases−related bacterial stains. In addition, the chemical profile of four of these essential oils with the most promising activity is being described for the first time.

## 2. Results

### 2.1. Antimicrobial Screening of Cerrado Plants Essential Oils

The essential oils from the leaves of seven aromatic plants were obtained by hydrodistillation using a Clevenger−type apparatus, in yields of 0.01 to 0.2%, on a weight basis. The oils were tested against standard strains and clinical isolates of multi−resistant bacteria related to foodborne diseases (Table 1), obtained at the Veterinary Hospital of the Universidade Federal de Mato Grosso do Sul (HV-UFMS).

The EOs of *C. squalida*, *C. sessiliflora* and *My. guianensis* showed good antimicrobial activity against the multi−resistant *Staphylococcus* sp. 841, isolated from cow’s milk with bovine mastitis, with emphasis on *C. squalida*, which showed the strongest activity (MIC of 7.80 µg·mL^−1^). The remaining oils were moderately active against this bacterium. All samples also proved moderately active against *Staphylococcus* sp. 873, as well as against the standard strains of *P. aeruginosa* and *S. aureus*, with MICs ranging from 500 to 125 µg·mL^−1^. Except for *C. squalida* and *S. guianensis*, all samples were moderately active against the standard *E. coli* strain. When evaluated against the β−lactamase producer *E. coli* and *Salmonella* Typhi 905 strains, the EOs of *Ma. guianensis* and *C. sessiliflora* also showed moderate activity, with an MIC of 500 µg·mL^−1^, while the remaining samples proved inactive against these bacteria.

### 2.2. Chemical Profile of the Bioactive Essential Oils

The chemical composition of the EOs with the best profiles of antimicrobial activity, namely *C. squalida*, *C. sessiliflora, My. guianensis* and *Ma. guianensis*, was analyzed by gas chromatography coupled to mass spectrometry (GC/MS) (Table 2). 

The analysis of the EOs obtained from *C. squalida* and *My. guianensis* revealed the presence of 40 and 28 components, respectively, mostly of the sesquiterpene−type, and with a predominance of hydrocarbon sesquiterpenes (78.04% and 91.36%, respectively). Among these compounds, viridiflorene (14.32%), germacrene D (12.74%), β−caryophyllene (12.54%) and δ−cadinene (11.60%) were the principal components of *C. squalida*, while isocaryophyllene (21.81%) followed by valencene (11.52%) and δ−cadinene (11.03%) were those of *My. guianensis*.

Twenty−two compounds were identified in the essential oil from the leaves of *Ma. guianensis,* which was shown to be composed almost exclusively of hydrocarbon sesquiterpenes (94.85%), such as bicyclogermacrene (31.32%), germacrene D (28.39%), β−caryophyllene (15.45%) and germacrene B (5.19%).

The EO of *C. sessiflora*, on the other hand, showed similar contents of mono− and sesquiterpenes (roughly 42% of each class), in which α−pinene was the predominant monoterpene (38.65%), while β−caryophyllene (13.35%) and aromadendrene (12.52%) were the main sesquiterpene−type components.

## 3. Discussion

*Chromolaena squalida*, *Campomanesia sessiliflora*, *Myrsine guianensis*, *Matayba guianensis*, *Siparuna guianensis*, *Ocotea minarum* and *Endlicheria paniculata* were selected for the screening of antimicrobial activity based on the criteria of large distribution/occurrence in the Cerrado biome, as well as organoleptic characteristics, focusing on plants with leaves that, when crushed, give off a remarkable fragrance. The species selected in the present study are representatives of the families Asteraceae, Myrtaceae, Primulaceae, Sapindaceae, Siparunaceae and Lauraceae.

Several bacteria are related to foodborne diseases, such as the commensal *S. aureus* and *E. coli*, diarrheagenic *E. coli*, other *Staphylococcus* species, *Clostridium botulinum*, *Salmonella enterica*, *Shigella* spp, *Campylobacter jejuni* and *Yersinia enterocolitica*, among others [25]. In the present work, standard strains of *S. aureus*, *P. aeruginosa*, *E. coli* and β−lactamase−producing *E. coli* were selected, in addition to two clinical isolated coagulase positive *Staphylococcus* spp. and *Salmonella* Typhi.

The EOs of *C. squalida*, *C. sessilifora* and *My. guianensis* showed the most potent antimicrobial activity against *Staphylococcus* sp. 841 resistant to ampicillin, doxycycline and clindamicyn, isolated from milk samples produced by cows affected by mastitis, with MIC values ranging from 7.80 to 31.25 µg·mL^−1^. Among these bioactive EOs, emphasis should be given to that from *C. squalida*, which strongly inhibited this bacterium with the lowest MIC, whose value is considered a good result even for pure substances [26]. All tested oils were moderately active (MICs 250 to 500 µg·mL^−1^) against *Staphylococcus* sp. 873, also isolated from milk, however showing a larger resistance profile (penicillins, including oxacillin, tetracyclines, lincosamides, quinolones, macrolides and second generation cephalosporines), as well as against the standard strain of *S. aureus* (MICs 125 to 500 µg·mL^−1^).

*Staphylococcus aureus* is an important causative agent of bovine mastitis, which is one of the most cost−intensive diseases in the dairy industry [27]. Furthermore, enterotoxigenic *S. aureus* strains have the potential to induce foodborne intoxications in humans transmitted by dairy products. A recent study evaluated over a thousand milk samples and revealed that *S. aureus* can effectively enter the dairy production chain via contaminated milk with subclinical *S. aureus* intramammary infections [28]. Outbreaks caused by staphylococci have been reported all over the word, from both developed and developing countries, such as USA, United Kingdom, France, Austria, Japan, China, Taiwan, Korea, Brazil and Argentina, among others [6,29], reinforcing the difficulty in containment and treating this pathogen.

It is notable that, regarding the chemical composition, the main components of the four EOs analyzed are mostly non oxygenated mono− and sesquiterpenes. In addition, the major components of *C. squalida* oil, with the best activity profile, are also present in the other EOs, however, in different proportions. The synergistic effect of these components in variable concentrations might therefore be overwhelming. Antimicrobial activity against *Staphylococcus aureus* and *Candida guilliermondii* was also reported for EOs rich in compounds similar to those found in *C. squalida*, such as EO from the leaves of *Duguetia gardneriana* (Annonaceae), with germacrene D (28.1%), viridiflorene (24.0%), β−pinene (12.6%), α−pinene (9.1%) and β−caryophyllene (5.6%) as its main constituents [30]. β−Caryophyllene, one of the main compounds in *C. squalida* oil, proved active against *Streptococcus mutans* [31], *Staphylococcus aureus* [32] and *Helicobacter pylori* [33]. Pieri et al. (2016) [34] evaluated β−caryophyllene against 32 microorganisms linked to bacterial dental plaque formation in dogs, of which, 24 were sensitive to this sesquiterpene at concentrations up to 100 mg·mL^−1^.

Evaluated against Gram−negative bacteria, which tend to be more resistant to antimicrobial agents than Gram−positive bacteria because of the additional protection afforded by the outer membrane [35], all EOs showed moderate activity against *P. aeruginosa* and *E. coli* (standard strain) (MICs 500 µg·mL^−1^), except for *C. squalida* and *S. guianensis*, which proved inactive against the latter.

It is worth mentioning that only *Ma. guianensis* EO showed activity against β−lactamase−producing *E. coli*, with an MIC of 500 µg·mL^−1^. The composition of this EO is characterized by the presence of sesquiterpenes, with no trace of monoterpenoids, of which 31.32% consist of bicyclogermacrene. This main component was previously reported as active against *P. aeruginosa, Acinetobacter baumanii* and *E. coli* [36].

The EO of *C. sessiliflora* was the only sample active against *Salmonella* Typhi 905, resistant to clindamicyn, penicillin and oxacillin, with an MIC of 500 µg·mL^−1^. A particular feature in the chemical profile of this EO was the presence of the monoterpene α−pinene as its major component (38.65% of the total oil composition). This monoterpene is listed by the US Food and Drug Administration among the food additives permitted for direct addition to food for human consumption, and also has been considered as a potentially useful natural antibacterial preservative as an alternative for SO_2_ in winemaking [37].

Although the antimicrobial effects and chemical composition of the plants evaluated in the present study have not been previously investigated, species belonging to the same genera as those investigated herein are known to exhibit antimicrobial properties. The EOs from flowers and fruits of *Chromolaena laevigata* were investigated for their chemical composition and antimicrobial activity, showing an MIC of 62.5 mg·mL^−1^ against *Candida albicans* and *S. aureus*, and an MIC of 500 mg·mL^−1^ against *P. aeruginosa* [38]. The EO from the leaves of *Campomanesia adamantium* showed high activity against *S. aureus, P. aeruginosa* and *C. albicans* and was moderately active against *E. coli*, and its main compounds were found to be monoterpenes, such as limonene, α−pinene and β−pinene [39]. The EO extracted from the leaves of *Campomanesia guazumifolia* (Cambess.) O. Berg strongly inhibited the strains of *S. aureus* (MIC 15 ± 0.1 µg·mL^−1^), *E. coli* (MIC 25 ± 0.2 µg·mL^−1^) and *C. albicans* (MIC 5 ± 0.1 µg·mL^−1^). Sixty−eight compounds were identified in its oil, of which the main constituents were bicyclogermacrene (15%), globulol (5%) and spathulenol (5%) [40].

The use of leaves as a source of EOs warrants the sustainable use of these plants. This fact deserves to be highlighted, since such plants have popular/medicinal uses. The decoction of leaves of *Chromolaena squalida*, popularly known as cambará−roxa, mata−pasto, casadinha or erva−de−são−miguel [41], is used in folk medicine to treat cough and as an antipyretic [42]. The species *Campomanesia sessiliflora*, popularly known as guabiroba−verde, is frequently found in the Brazilian Cerrado [43] and produces edible fruits, consumed in natura by the local population. *Matayba guianensis*, known as brazeiro, camboatá, camboatã, camboatá−branco, olho−de−cotia, mataíba, batabaíba, cuvantã, jatuá−uba, jatuá−iba, pau−da−digestão, atou−aou, tou−aou or canela−de−negro [44], is considered a medicinal plant, and infusions and decocts of its root are used in the treatment of respiratory diseases and to alleviate back and leg pain [45]. The plant *Mysrine guianensis*, popularly known as caapororoca, capororoca and pororoca [46], is used in folk medicine as an antiseptic [47] and antiparasitic [48].

Food safety can be considered as a priority, since foodborne diseases have emerged as a serious public health problem, besides leading to huge economic and social losses worldwide, especially in low− and middle−income countries. Our results reveal a promising activity of EOs from Cerrado plants against foodborne bacteria, revealing their potentialities for the development of new agents to prevent the spreading of resistant bacterial contamination in the food production chain, contributing to reduce the risks of foodborne diseases. Further toxicological studies are required for safety purposes.

## 4. Materials and Methods

**Plant Material**. The leaves of the species *Chromolaena squalida* (DC.) R.M. King & H. Rob. (Asteraceae), *Campomanesia sessiliflora* (O. Berg) Mattos (Myrtaceae), *Matayba guianensis* Aubl. (Sapindaceae), *Myrsine guianensis* (Aubl.) Kuntze (Primulaceae), *Siparuna guianensis* A. DC. (Siparunaceae), *Ocotea minarum* (Nees & Mart.) Mez (Lauraceae) and *Endlicheria paniculata* (Spreng.) J.F. Macbr. (Lauraceae) were collected in March 2020, at Campo Grande, Mato Grosso do Sul, Brasil (20°30’29”S and 54°36’58”W). The botanical identification of the species was carried out by Dr. Flávio M. Alves (UFMS), and all exsiccates (deposit number are 2040, 37574, 74331, 74300, 74339, 74374, 72774, respectively) were deposited at the CGMS Herbarium (UFMS). License for research on Brazil’s biodiversity, #A1D8864.

**Essential oil extraction**. Fresh leaves of *C. squalida* (526 g), *C. sessiliflora* (486 g), *Ma. guianensis* (576 g), *My. guianensis* (483 g), *S. guianensis* (487 g), *O. minarum* (500 g) and *E. paniculata* (424 g) were separately subjected to hydrodistillation in a Clevenger−type apparatus for 8 h, to yield 280 mg (0.05% *w*/*w*), 916 mg (0.19% *w*/*w*), 1.2 g (0.2% *w*/*w*), 76 mg (0.015% *w*/*w*), 2.6 g (0.5% *w*/*w*), 690 mg (0.14% *w*/*w*) and 544 mg (0.13% *w*/*w*) of essential oils, respectively.

**Gas chromatography/mass spectrometry (GC/MS)**. The GC/MS analysis was performed using a Shimadzu GC/MS QP−2010 PLUS Gas Chromatograph coupled to a mass spectrometer operating at 70 eV and an Rtx−MS column (Restek^®^, Bellefonte, PA, USA) (30 m × 0.25 mm × 0.25 pm) consisting of 5%−diphenyl−95%−dimethylpolysiloxane.

**Chromatography conditions.** Injector temperature was 250 °C, with the carrier gas (helium) at a flow rate of 1 mL·min^−1^, pressure of 87.1 kPa and column oven temperature programmed to 50–260 °C (3 °C/min). A mixture of linear hydrocarbons (C9 to C22 alkanes) was injected under the same experimental conditions. The identification of the constituents in the essential oil was performed by comparing the mass spectra obtained with those of the equipment database (Wiley 7 lib and Nist 08 lib) and by using the retention index (RI), calculated for each constituent as previously described [24].

**Antimicrobial assays.** All reagents and media for the antibacterial assays were purchased from Sigma Aldrich™. The standard bacterial strains used were *S. aureus* (NEWP0023), *E. coli* (NEWP0022), *E. coli* (NEWP0018, β−lactamase producer) and *P. aeruginosa* (NEWP0027). Veterinary clinical strains *Staphylococcus* sp. 841(resistant to ampicillin, doxycycline, clindamicyn, cefoxitin), *Staphylococcus* sp. 873 (resistant to ampicilin, doxycycline, clindamicyn, penicillin, oxacillin, norfloxacin, cefoxitin, azithromycin) and *Salmonella* Typhi 905 (resistant to clindamicyn, penicillin, oxacillin) were provided by the Veterinary Hospital, Faculty of Veterinary Medicine and Animal Science of Universidade Federal de Mato Grosso do Sul (Campo Grande, Brazil). License of the bacterial collection for research on Brazil’s biodiversity, #C69392D.

The antimicrobial activity of the essential oils was determined by the broth microdilution method, as described by Manda et al. (2018) [49]. Two−fold dilutions were performed in 96−well plates prepared with Mueller–Hinton broth to reach a final concentration of 1.95 to 4000 μg·mL^−1^, with a 100 μL final volume in each well. The inoculums were overnight cultures of each bacterial species in Mueller–Hinton agar diluted in sterile saline solution (0.45%) to a concentration of approximately 10^8^ CFU·mL^−1^. This solution was diluted 1/10 in saline solution (0.45%) and 5 μL was added to each well containing the test samples. All experiments were performed in triplicate and the microdilution trays were incubated at 36 °C for 18 h. Then, 20 μL of an aqueous solution (0.5%) of triphenyl tetrazolium chloride (TTC) was added to each well and the trays were again incubated at 36 °C for 2 h. In those wells where bacterial growth did occur, TTC changed from colorless to red. MIC was defined as the lowest concentration of each substance at which no color change occurred and was expressed in μg·mL^−1^.

## Figures and Tables

**Table 1:** Minimum inhibitory concentration values (MIC, µg·mL^−1^) of the essential oils obtained from the leaves of *Chromolaena squalida*, *Campomanesia sessiliflora*, *Myrsine guianensis*, *Matayba guianensis*, *Siparuna guianensis*, *Ocotea minarum* and *Endlicheria paniculata* against standard strains of *S. aureus*, *E. coli* and *P. aeruginosa*, and clinical isolated resistant strains *.

	MIC (µg·mL^−1^)						
Plant species/Essential Oil	*E. coli* NEWP0022	*E. coli* NEWP 0018	*S. aureus* NEWP 0023	*P. aeruginosa* NEWP0027	*Staphylococcus* sp. 841	*Staphylococcus* sp. 873	*Salmonella* Typhi 905
*Chromolaena squalida*	>1000	>1000	125	500	7.80	250	>1000
*Campomanesia sessiliflora*	500	>1000	250	500	31.25	250	500
*Myrsine guianensis*	500	>1000	500	500	31.25	500	>1000
*Matayba guianensis*	500	500	500	500	125	500	>1000
*Siparuna guianensis*	>1000	>1000	500	500	500	500	>1000
*Ocotea minarum*	500	>1000	250	500	250	250	>1000
*Endlicheria paniculata*	500	>1000	500	500	500	250	>1000
Gentamincin	≤0.5	≤0.5	≤0.5	3.5	≤0.5	3.5	≤0.5

* Resistance profiles: *E. coli* NEWP0018: β−lactamase producer. Coagulase positive *Staphylococcus* sp. 841: resistant to ampicillin, doxycycline, clindamycin, cefoxitin. Coagulase positive *Staphylococcus* sp. 873: resistant to ampicillin, doxycycline, clindamycin, penicillin, oxacillin, norfloxacin, cefoxitin, azithromycin. *Salmonella* Typhi 905: resistant to clindamycin, penicillin, oxacillin.

**Table 2:** Chemical composition of the bioactive essential oils from Cerrado species.

Compounds	Molecular Formula	RI ^+^_Exp_	RI ^++^_Ref._	*Chromolaena squalida*	*Campomanesia sessiliflora*	*Myrsine* *guianensis*	*Matayba guianensis*
				Peak Area (%)			
α-Thujene **1**	C_10_H_16_	922	924	0.27	0.27	0.01	-
α-Pinene **2**	C_10_H_16_	930	932	1.00	38.65	-	-
Camphene **3**	C_10_H_16_	944	946	-	0.03	-	-
Sabinene **4**	C_10_H_16_	968	969	0.06	-	-	-
β-Pinene **5**	C_10_H_16_	972	974	1.38	0.69	-	-
Myrcene **6**	C_10_H_16_	986	988	1.35	-	-	-
α-Phellandrene **7**	C_10_H_16_	1002	1002	0.04	0.20	-	-
α-Terpinene **8**	C_10_H_16_	1013	1014	0.10	-	-	-
o-Cymene **9**	C_10_H_14_	1021	1022	0.14	0.10	-	-
Limonene **10**	C_10_H_16_	1024	1024	2.18	2.10	-	-
(*Z*)-β-Ocimene **11**	C_10_H_16_	1033	1032	0.14	-	-	-
(*E*)-β-Ocimene **12**	C_10_H_16_	1043	1044	4.77	-	-	-
γ-Terpinene **13**	C_10_H_16_	1054	1054	0.30	0.17	-	-
Terpinolene **14**	C_10_H_16_	1085	1086	0.76	0.07	-	-
Eucalyptol **15**	C_10_H_18_O	1027	1026	-	2.74	0.02	-
Linalool **16**	C_10_H_18_O	1097	1095	0.15	1.67	0.05	-
Terpinen-4-ol **17**	C_10_H_18_O	1173	1174	0.36	0.04	-	-
α-Terpineol **18**	C_10_H_18_O	1188	1186	0.19	0.37	-	-
δ-Elemene **19**	C_15_H_24_	1332	1335	0.91	-	-	2.73
α-Cubebene **20**	C_15_H_24_	1344	1348	0.36	-	0.44	0.14
Cyclosativene **21**	C_15_H_24_	1362	1369	0.22	-	-	-
α-Ylangene **22**	C_15_H_24_	1366	1373	0.37	-	0.59	0.24
Isoledene **23**	C_15_H_24_	1368	1374	0.19	0.32	-	0.47
α-Copaene **24**	C_15_H_24_	1371	1374	1.95	0.11	7.00	1.34
β-Bourbonene **25**	C_15_H_24_	1380	1387	0.32	-	0.30	0.21
β-Elemene **26**	C_15_H_24_	1387	1389	1.26	0.19	0.55	1.95
α-Gurjunene **27**	C_15_H_24_	1405	1409	0.31	1.08	0.06	0.21
Isocaryophyllene **28**	C_15_H_24_	1414	1408	-	-	21.81	-
β-Caryophyllene **29**	C_15_H_24_	1415	1417	12.54	13.35	-	15.45
Aromadendrene **30**	C_15_H_24_	1434	1439	0.32	12.52	7.82	1.28
α-Humulene **31**	C_15_H_24_	1448	1452	1.92	1.69	7.80	2.10
(*E*)-β-Farnesene **32**	C_15_H_24_	1452	1454	-	-	0.68	-
α-Patchoulene **33**	C_15_H_24_	1456	1454	1.26	3.57	0.77	0.81
10-β-Cadina-1(6),4-diene **34**	C_15_H_24_	1468	1475	1.38	-	-	-
α-Amorphene **35**	C_15_H_24_	1473	1483	-	-	-	1.37
Germacrene D **36**	C_15_H_24_	1478	1480	12.74	-	1.95	28.39
β-Selinene **37**	C_15_H_24_	1482	1489	0.65	0.47	3.51	0.33
γ-Muurolene **38**	C_15_H_24_	1482	1478	4.85	0.42	7.05	-
Valencene **39**	C_15_H_24_	1490	1496	-	-	11.52	-
Viridiflorene **40**	C_15_H_24_	1490	1496	14.32	7.55	-	-
Bicyclogermacrene **41**	C_15_H_24_	1493	1500	-	-	0.37	31.32
α-Muurolene **42**	C_15_H_24_	1496	1500	3.29	-	0.64	0.56
γ-Cadinene **43**	C_15_H_24_	1510	1513	4.06	0.77	4.45	-
δ-Cadinene **44**	C_15_H_24_	1519	1522	11.60	-	11.03	0.76
*trans*-Cadina-1,4-diene **45**	C_15_H_24_	1527	1533	0.73	-	-	-
α-Calacorene **46**	C_15_H_24_	1539	1544	-	0.10	-	-
Germacrene B **47**	C_15_H_24_	1552	1559	2.49	-	3.02	5.19
Elemol **48**	C_15_H_26_O	1545	1548	-	0.14	-	-
Spathulenol **49**	C_15_H_24_O	1574	1577	-	6.46	2.21	2.29
(*E*)-Nerolidol **50**	C_15_H_26_O	1559	1561	-	-	4.71	-
Viridiflorol **51**	C_15_H_26_O	1598	1592	8.74	1.44	0.89	1.83
5-epi-7-epi-α-Eudesmol **52**	C_15_H_26_O	1598	1607	-	-	-	0.67
γ-Eudesmol **53**	C_15_H_26_O	1628	1630	-	0.59	-	-
epi-α-Muurolol **54**	C_15_H_26_O	1637	1640	-	0.62	-	-
δ-cadinol **55**	C_15_H_26_O	1643	1644	-	0.12	0.48	-
β-Eudesmol **56**	C_15_H_26_O	1649	1649	-	1.36	0.19	-
	Identified compounds (%)			99.97	99.97	99.92	99.64
	Monoterpene Hydrocarbons (%)			12.49	42.28	0.01	-
	Oxygenated Monoterpenes (%)			0.70	4.82	0.07	-
	Sesquiterpene Hydrocarbons (%)			78.04	42.14	91.36	94.85
	Oxygenated Sesquiterpenes (%)			8.74	10.73	8.48	4.79

The compounds are listed in order of their elution from the Rtx-MS column. LRI: retention indexes on the Rtx-MS column (relative to n-alkanes C_9_-C_20_). ^+^ Experimental retention index. ^++^ Retention index from the literature [24].
